# Risk factors for breast cancer in a cohort of mammographic screening program: a nested case–control study within the FRiCaM study

**DOI:** 10.1002/cam4.1427

**Published:** 2018-04-14

**Authors:** Francesca Bravi, Adriano Decarli, Antonio Giampiero Russo

**Affiliations:** ^1^ Department of Clinical Sciences and Community Health Università degli Studi di Milano Milan Italy; ^2^ Epidemiology Unit Agency for Health Protection of Milan Milan Italy

**Keywords:** Breast cancer, cohort, nested case–control study, prevention, risk factors

## Abstract

Breast cancer is the most common cancer diagnosis and the leading cause of cancer death among women in the world, and differences across populations indicate a role of hormonal, reproductive and lifestyle factors. This study is based on a cohort of 78,050 women invited to undergo a mammogram by Local Health Authority of Milan, between 2003 and 2007. We carried out a nested case–control study including all the 3303 incident breast cancer cases diagnosed up to 2015, and 9909 controls matched by age and year of enrollment. Odds ratios (ORs) and corresponding 95% confidence intervals (CIs) were estimated using logistic regression models. The ORs were 0.88 (95% CI: 0.78–0.98) for an age at menarche ≥14 years and 1.39 (95% CI: 1.07–1.81) for an age of 30 years or older at first pregnancy. Body mass index (BMI) was positively associated with breast cancer risk in women older than 50 years (OR = 1.89, 95% CI: 1.54–2.31, for BMI≥30 vs. <20), while the association tended to be inverse in younger women. A high mammographic density increased breast cancer risk (OR = 2.61, 95% CI: 2.02–3.38 for density >75% vs. adipose tissue). The ORs were 1.67 (95% CI: 1.47–1.89) and 2.04 (95% CI: 1.38–3.00) for one first‐degree relative and two or more relatives affected by breast cancer, respectively. Our study confirms the role of major recognized risk factors for breast cancer in our population and provides the basis for a stratification of the participants in the mammographic screening according to different levels of risk.

## Introduction

Breast cancer is the most common cancer diagnosis and the leading cause of cancer death among women in the world, with 1.7 million cases and 521,900 deaths estimated in 2012 1. The shape of age‐specific incidence rates is similar across countries, although large differences exist in the absolute rates at every age, with fourfold variations between Western Europe and Asian regions. Furthermore, some studies reported that migrants assume the rates of the hosting populations in the following generations 2, 3. These patterns are compatible with differences across populations in hormonal and reproductive factors, as well as lifestyle habits.

An early age at menarche—which implies the onset of a mature hormonal pattern, including onset of ovulation, cyclic hormonal changes, and menstruation—has been consistently related to an increased risk of breast cancer, with an estimated 5% of increase in risk for every year of menarche anticipation 4, 5, 6. Alike, a late age at menopause—which marks the cessation of ovulation and hormonal cycles—has been positively associated with breast cancer risk 4, 5, 6. Nulliparous women have an increased risk of breast cancer, compared to parous ones, and among the latter, a younger age at first full‐term pregnancy is associated with a lower risk 5, 6. All the aforementioned aspects point to a role of the lifetime “dose” of exposure to endogenous hormones.

Another well‐established risk factor for breast cancer is a family history of the disease in first‐degree relatives, the risk being further increased in the presence of more than one affected relative 5, 6, 7.

Among lifestyle risk factors, alcohol drinking has been consistently related to breast cancer, with an increase in risk of about 20% for moderate drinkers and of 60% for heavy drinkers 5, 6, 8, 9, while the role of diet appears modest 9, with a weak inverse association with fiber intake 5, 6, 10, fruit, and fruit and vegetables combined, but not vegetables alone 5, 6, 11, and a weak positive association with total fat, if any 5, 6, 12.

In this study, we evaluated the role of hormonal factors, lifestyle habits, and family history of breast cancer, using data from a large cohort of women invited to participate to a mammographic screening program in Italy. To our knowledge, this is the first study that quantified these associations in an Italian population participating to an organized a mammographic screening program.

## Materials and Methods

### Study population

This study is based on data from a cohort of 78,050 women, aged between 41 and 76 years, resident in the municipality of Milan, who were invited to participate in the mammographic screening program of the Local Health Authority of Milan from 2003 to 2007 in the framework of a study with the acronym of FRiCaM (Risk Factors for Breast Cancer: *F*attori di *Ri*schio per il *Ca*rcinoma della *M*ammella), supported by a specific grant of the Italian League of Cancer Prevention.

All participants signed an informed consent and completed a self‐administered questionnaire, developed with the aim to identify women at high risk of breast cancer, who may benefit from a high‐intensity screening program.

A total of 131,246 women received a questionnaire while waiting for the mammographic exam: 71,398 of them completed the questionnaire (54%).

In order to obtain information from a sample of nonscreened women, 20,000 questionnaires were sent by mail to nonattenders to a mammographic screening: 6652 women, who did not adhere to the mammographic screening, accepted to provide the questionnaire through postal delivery (33%).

The questionnaire requested detailed information on socio‐demographic characteristics, anthropometric measures, menstrual and reproductive history, health status, family history of cancer (including breast), and lifestyle factors, including dietary habits. Mammographic density was available only for the sample of screened women and was read by experienced screening radiologists and classified in three categories: (1) almost entirely fat (less than 25 percent glandular), (2) scattered fibroglandular densities or heterogeneously dense (approximately 25–75% glandular), and (3) extremely dense (more than 75% glandular).

As all study participants provided a written informed consent together with the questionnaire, the date of the signing of the consensus was considered as the date of enrollment in the study. Breast cancer cases were identified through record linkage between the cohort and the Cancer Registry of Milan, which is active since 1999. This allowed to identify 3532 breast cancer cases diagnosed up to 2015. Among these, we excluded 229 women who had a date of diagnosis preceding the date of enrollment in the study. Thus, the final number of breast cancer cases included in this study is 3303. The study was approved by the Ethics Committee of the Local Health Authority of Milan. Record linkage with the Cancer Registry of Milan and with Civil Registry allowed to assess vital status and absence of cancer among the controls at the end of the follow‐up period.

### Nested case–control study

Within the screening cohort, we carried out a nested case–control study. All the 3303 incident breast cancer cases were included. For each case, three control subjects were chosen at random among cohort members alive and free of breast cancer, individually matched to cases by age (in quinquennia) and year of enrollment in the study. Thus, the present work is based on 3303 incident cases of breast cancer and 9909 matched controls.

### Statistical analysis

We estimated odds ratios (ORs) and corresponding 95% confidence intervals (CIs) of breast cancer for the selected risk factors, through logistic regression models conditioned on age in quinquennia and year of enrollment, and adjusted for education (no/primary school, secondary/vocational school, high school, university), marital status (married/cohabitant, separated/divorced, widow, never married), body mass index (<20, 20–24.9, 25–29.9, ≥30 kg/m^2^), alcohol drinking (never, >0–<1 drink/week, 1 drink/week–<1 drink/day, 1 drink/day, >1 drink/day), age at menarche (≤11, 12–13, ≥14), age at first birth (<20, 20–24, 25–29, ≥30), menopausal status (premenopause, postmenopause), family history of breast cancer (no, yes), when appropriate (i.e., when the factor was not considered as exposure variable). Women with a missing value for an exposure variable were excluded from the analysis of that variable, while women with missing values on a confounding variable were included in the analyses, using a separate category for missing values. None of the confounding variables had missing values for more than 5% of both cases and controls. Regression analyses with ordered categories were used to test the presence of a linear trend.

All the analyses were performed using the SAS software, version 9.4 (SAS Institute, Inc., Cary, NC, USA).

## Results

Table 1 shows the distribution of baseline socio‐demographic characteristics of 3303 breast cancer cases and 9909 matched participants in the FRiCaM study. Cases tended to be more educated and were more likely never married, as compared to controls.

**Table 1:** Socio‐demographic characteristics, and corresponding odds ratios (ORs) and 95% confidence intervals (CIs) of the participants in the FRiCaM study

	Cases *N* (%)	Controls *N* (%)	Crude OR (95% CI)
Age			
<50	125 (3.78)	375 (3.78)	—
50–54	549 (16.62)	1647 (16.62)	—
55–59	732 (22.16)	2196 (22.16)	—
60–64	872 (26.40)	2616 (26.40)	—
65–69	700 (21.19)	2100 (21.19	—
≥70	325 (9.84)	975 (9.84)	—
Year of enrollment			
2003	787 (23.83)	2361 (23.83)	—
2004	1558 (47.17)	4674 (47.17)	—
2005	874 (26.46)	2622 (26.46)	—
2006	68 (2.06)	204 (2.06)	—
2007	16 (0.48)	48 (0.48)	—
Education^a^			
No/primary school	536 (16.48)	2070 (21.28)	1.00^b^
Secondary/vocational school	1380 (42.42)	3917 (40.27)	1.37 (1.23–1.54)
High school	831 (25.55)	2335 (24.01)	1.40 (1.23–1.58)
University	506 (15.55)	1404 (14.44)	1.42 (1.23–1.64)
Marital status^a^			
Married/cohabitant	2155 (67.41)	6657 (69.34)	1.00^b^
Separated/divorced	289 (9.04)	876 (9.12)	1.02 (0.89–1.18)
Widow	427 (13.36)	1311 (13.65)	1.01 (0.89–1.14)
Never married	326 (10.20)	757 (7.88)	1.33 (1.16–1.53)

a The sum does not add up to the total because of missing values.

b Reference category.

Table 2 provides the distribution of reproductive factors, health status and family history of breast cancer, and corresponding 95% ORs and CIs, among participants in the FRiCaM study. A higher age at menarche was associated with a reduced risk of breast cancer (OR = 0.88, 95% CI: 0.78–0.98 for ≥14 years as compared to ≤11 years), with a significant trend of decrease in risk (*P* = 0.0265). A higher age at first live birth was associated with an increased risk of breast cancer (OR = 1.39, 95% CI: 1.07–1.81 for ≥30 years vs. <20 years), with a significant trend in risk (*P* = 0.0006). Previous breast biopsies were associated with an increase in breast cancer risk (OR = 1.86, 95% CI: 1.63–2.12). Mammographic density was associated with breast cancer risk, the ORs being 1.59 (95% CI: 1.31–1.93) for a density <25%, 2.25 (95% CI: 1.86–2.73) for 25–75%, and 2.61 (95% CI: 2.02–3.38) for >75%, as compared to almost entirely adipose tissue (*P* < 0.0001). Among women older than 50 years, cases were more frequently overweight (OR = 1.68, 95% CI: 1.40–2.01) or obese (OR = 1.89, 95% CI: 1.54–2.31) as compared to women with a body mass index (BMI) <20 (*P* for trend <0.0001), while among women younger than 50 years BMI tended to be inversely related to breast cancer, though in the absence of statistical significance. Women who experienced a previous breast surgery had an increased risk of breast cancer (OR = 2.01, 95% CI: 1.75–2.29). Family history of breast cancer in first‐degree relatives was associated with an increased risk of breast cancer, the ORs being 1.67 (95% CI: 1.47–1.89) for women with one relative affected by breast cancer and 2.04 (95% CI: 1.38–3.00) for women with two or more relatives affected by breast cancer (*P* for trend <0.0001). No significant association was evident for the other considered factors, including menopausal status, oral contraceptives use, hormone replacement therapy use, previous diagnosis of other cancers, previous ovarian surgery, previous Smear test, and previous mammography.

**Table 2:** Distribution of reproductive factors, health status and family history of breast cancer, and corresponding odds ratios (ORs) and 95% confidence intervals (CIs) among the participants in the FRiCaM study

	Cases *N* (%)	Controls *N* (%)	OR (95% CI)^a^
Age at menarche (years)^b^			
≤11	827 (25.66)	2355 (24.39)	1.00^c^
12–13	1641 (50.92)	4750 (49.19)	1.00 (0.90–1.10)
≥14	755 (23.43)	2552 (26.43)	**0.88 (0.78–0.98)**
*P* for trend			0.0265
Age at first live birth (years)^b^			
<20	83 (3.23)	337 (4.14)	1.00^c^
20–24	751 (29.23)	2635 (32.36)	1.14 (0.88–1.47)
25–29	1077 (41.92)	3375 (41.44)	1.23 (0.95–1.60)
≥30	658 (25.61)	1797 (22.07)	**1.39 (1.07–1.81)**
*P* for trend			0.0006
Menopausal status			
Premenopause	736 (22.28)	2191 (22.11)	1.00^c^
Postmenopause	2567 (77.72)	7718 (77.89)	0.97 (0.86–1.10)
Oral contraceptives use^b^			
Never	2003 (65.18)	6059 (65.74)	1.00^c^
Ever	1070 (34.82)	3158 (34.26)	1.00 (0.91–1.10)
Hormone replacement therapy^b^			
Never	2286 (73.72)	6717 (72.84)	1.00^c^
Ever	815 (26.28)	2504 (27.16)	1.06 (0.96–1.17)
Previous breast biopsies^b^			
No	2758 (87.28)	8817 (92.91)	1.00^c^
Yes	402 (12.72)	673 (7.09)	**1.86 (1.63–2.12)**
Mammographic density^b^			
Adipose	153 (5.15)	817 (9.00)	1.00^c^
<25%	786 (26.45)	2780 (30.61)	**1.59 (1.31–1.93)**
25–75%	1160 (39.03)	3058 (33.67)	**2.25 (1.86–2.73)**
>75%	186 (6.26)	446 (4.91)	**2.61 (2.02–3.38)**
*P* for trend			<0.0001
BMI in women ≤50 years (kg/m^2^)^b^			
<20	39 (15.54)	107 (13.99)	1.00^a^
20–24.9	138 (54.98)	400 (52.29)	0.90 (0.59–1.38)
25–29.9	56 (22.31)	187 (24.44)	0.75 (0.46–1.24)
≥30	18 (7.17)	71 (9.28)	0.70 (0.36–1.38)
*P* for trend			0.1803
BMI in women >50 years (kg/m^2^)^b^			
<20	182 (6.12)	797 (8.94)	1.00^a^
20–24.9	1414 (47.51)	4257 (47.76)	**1.51 (1.27–1.80)**
25–29.9	972 (32.66)	2771 (31.09)	**1.68 (1.40–2.01)**
≥30	408 (13.71)	1088 (12.21)	**1.89 (1.54–2.31)**
*P* for trend			<0.0001
Previous diagnosis of other cancers^b^			
No	2807 (94.70)	8397 (95.59)	1.00^c^
Yes	157 (5.30)	387 (4.41)	1.19 (0.98–1.44)
Previous breast surgery^b^			
No	2744 (87.17)	8823 (93.29)	1.00^c^
Yes	404 (12.83)	635 (6.71)	**2.01 (1.75–2.29)**
Previous ovarian surgery^b^			
No	2530 (85.16)	7543 (84.82)	1.00^c^
Yes	441 (14.84)	1350 (15.18)	0.98 (0.87–1.10)
Previous Smear test^b^			
No	2963 (93.06)	623 (6.55)	1.00^c^
Yes	221 (6.94)	8884 (93.45)	0.94 (0.80–1.11)
Previous mammography^b^			
No	135 (4.19)	432 (4.47)	1.00^c^
Yes	3088 (95.81)	9241 (95.53)	1.04 (0.85–1.27)
Number of first‐degree relatives affected by breast cancer			
0	2817 (85.29)	9003 (90.86)	1.00^c^
1	443 (13.41)	838 (8.46)	**1.67 (1.47–1.89)**
≥2	43 (1.30)	68 (0.69)	**2.04 (1.38–3.00)**
*P* for trend			<0.0001

a Estimated through logistic regression models conditioned on age and year of enrollment, and adjusted for education, marital status, body mass index, alcohol drinking, age at menarche, age at first birth, menopausal status, family history of breast cancer, when appropriate. Statistically significant estimates are shown in bold.

b The sum does not add up to the total because of missing values.

c Reference category.

Table 3 describes the association between lifestyle risk factors and breast cancer. Alcohol drinking was associated with an increased risk of breast cancer, the OR being 1.21 (95% CI: 1.06–1.37) for >1 drink/week, as compared to never drinking (*P* for trend <0.0001). An inverse association was evident for fruit consumption (OR = 0.70, 95% CI: 0.53–0.93 for >1 fruit per day vs. <1 fruit per week, *P* for trend = 0.0170). No significant association emerged for the remaining considered lifestyle factors, including tobacco smoking, consumption of vegetables, cheese, red and white meat, and fish.

**Table 3:** Distribution of lifestyle risk factors, and corresponding 95% odds ratios (ORs) and confidence intervals (CIs) among the participants in the FRiCaM study

	Cases *N* (%)	Controls *N* (%)	OR (95% CI)^a^
Alcohol drinking (drinks/week)^b^			
Never	508 (15.88)	1671 (17.44)	1.00^c^
Occasional (>0–<1/week)	600 (18.76)	2056 (21.46)	0.91 (0.79–1.04)
1/week–<1/day	741 (23.17)	2239 (23.37)	1.03 (0.90–1.17)
1/day	388 (12.13)	1104 (11.52)	1.16 (1.00–1.36)
>1/day	961 (30.05)	2510 (26.20)	**1.21 (1.06–1.37)**
*P* for trend			<0.0001
Tobacco smoking^b^			
Never	1834 (57.08)	5338 (55.90)	1.00^c^
Ex	707 (22.00)	2144 (22.45)	0.91 (0.82–1.00)
Current	672 (20.92)	2067 (21.65)	0.92 (0.83–1.02)
*P* for trend			0.0681
Dietary habits			
Vegetable intake^b^			
<1/week	50 (1.56)	159 (1.65)	1.00^c^
1–6/week	768 (23.91)	2390 (24.82)	1.00 (0.72–1.39)
1/day	1014 (31.57)	2981 (30.96)	1.01 (0.73–1.41)
>1/day	1380 (42.96)	4098 (42.56)	0.97 (0.70–1.35)
*P* for trend			0.5829
Fruit intake^b^			
<1/week	73 (2.29)	167 (1.75)	1.00^c^
1–6/week	381 (11.96)	1105 (11.56)	0.75 (0.56–1.02)
1/day	717 (22.50)	2050 (21.45)	0.76 (0.57–1.02)
>1/day	2015 (63.25)	6235 (65.24)	**0.70 (0.53–0.93)**
*P* for trend			0.0170
Cheese intake^b^			
<1/week	219 (7.06)	696 (7.41)	1.00^c^
1–3/week	1674 (54.00)	5054 (53.82)	1.04 (0.89–1.23)
4–6/week	540 (17.42)	1565 (16.67)	1.07 (0.89–1.28)
≥1/day	667 (21.52)	2075 (22.10)	1.01 (0.85–1.21)
*P* for trend			0.8673
Red meat intake^b^			
<1/week	810 (25.67)	2426 (25.77)	1.00^c^
1/week	1013 (32.10)	3022 (32.10)	1.01 (0.90–1.12)
2–3/week	1007 (31.91)	3113 (33.07)	0.97 (0.87–1.08)
≥4/week	326 (10.33)	852 (9.05)	1.12 (0.96–1.31)
*P* for trend			0.5767
White meat intake^b^			
<1/week	395 (12.48)	1300 (13.68)	1.00^c^
1/week	858 (27.11)	2664 (28.03)	1.06 (0.93–1.22)
2–3/week	1508 (47.65)	4327 (45.53)	1.14 (1.00–1.30)
≥4/week	404 (12.76)	1212 (12.75)	1.09 (0.92–1.28)
*P* for trend			0.1101
Fish intake^b^			
<1/week	938 (29.34)	2865 (29.87)	1.00^c^
1/week	1223 (38.25)	3674 (38.30)	1.01 (0.91–1.11)
2–3/week	874 (27.34)	2625 (27.37)	0.99 (0.89–1.10)
≥4/week	162 (5.07)	428 (4.46)	1.10 (0.90–1.34)
*P* for trend			0.7700

a Estimated through logistic regression models conditioned on age and year of enrollment, and adjusted for education, marital status, body mass index, alcohol drinking, age at menarche, age at first birth, menopausal status, family history of breast cancer, when appropriate. Statistically significant estimates are shown in bold.

b The sum does not add up to the total because of missing values.

c Reference category.

## Discussion

In the present study, we carried out a comprehensive assessment of the role of several hormonal and lifestyle factors for breast cancer, using data from a large cohort of women participating in a mammographic screening program in Italy. We observed a 12% reduced risk of developing breast cancer for women who had the menarche at 14 years old or after, and a 39% increased risk for women who had their first pregnancy at 30 years old or after. These results are in line with those from previous studies 4, 5, 6 and confirm the hormonal mechanisms implied in the onset of breast cancer. Age at menarche is related to the onset of ovulation, the later the age at menarche, the later the onset of ovulation and, possibly, of regular menstrual cycles 5, 13. Moreover, some studies reported that hormonal levels throughout the whole reproductive years tend to be higher among women who have an earlier menarche 5, 14. Independent of parity, an earlier age at full‐term pregnancy is associated with a reduced risk of breast cancer 5, 6. After menarche and before the first pregnancy, the breast has relatively undifferentiated ducts and alveolar buds, named lobule types 1 and 2. Then, the glandular epithelial cells gradually differentiate into lobule types 3 and 4. The differentiation happens largely after the first pregnancy, and partially after subsequent pregnancies. Thus, when the first pregnancy occurs earlier, fewer cells are likely to have initiated the differentiation 5, 6. These results confirm that lifetime exposure to hormones is involved in the development of the disease, the longer the hormonal exposure, the higher the risk of breast cancer 15.

As for the role of body weight on breast cancer risk, it was dependent on age—as a proxy of menopausal status—in our, as well as in previous studies 5, 6, the association being inverse in premenopausal and positive in postmenopausal women. It has been suggested that in premenopause, obese women are less likely to ovulate, thus having reduced levels of circulating hormones. Conversely, after menopause, circulating estrogens derive mainly from adipose tissues, thus the higher the BMI, the higher the hormonal levels.

Mammographic density—that is, the overall percentage of dense tissue observed in the mammogram—has been consistently related to breast cancer risk and is modulated by hormonal, reproductive, and lifestyle risk factors associated with the disease. Our observation of an increased risk of breast cancer associated with a higher mammographic density is in agreement with previous studies 5, 6, 16.

Our study also confirmed that breast cancer risk is higher among women with a family history of breast cancer, the increase in risk being 67% among women with a first‐degree relative affected, and twofold in women with more than one relative affected by breast cancer. Several studies have reported this association, which may be due both to the fact that the relatives tend to be exposed to the same environmental and lifestyle factors, as well as to the fact that they share inheritable genetic susceptibility.

Considering lifestyle factors, we observed an increased risk of breast cancer among regular alcohol drinkers, which has been reported previously 5, 6, 8. Different mechanisms have been proposed to explain this association, including a direct carcinogenic effect of alcohol metabolites, an increase in circulating hormone levels, and an indirect effect as antagonist of folate 17, 18. In our study, the increase in risk was 21% among drinkers of more than one drink per day, and 16% among drinkers of one drink per day. Given the high prevalence of moderate alcohol drinkers in our population, even a small increase in risk may be a relevant public health concern.

We did not observe any significant role of dietary habits, with the only exception of fruit consumption, which was inversely related to breast cancer risk. Although several studies have been carried out on the role of dietary habits on breast cancer etiology, this is still an open issue, and the evidence on specific foods or nutrients is controversial, with weak and inconsistent associations reported, if any 9, 19. However, this finding may also be due to uncontrolled confounder, for example, physical activity, which is inversely related to breast cancer and is likely correlated with fruit intake 9.

Among the strengths of the present study, there are the population‐based design, the large sample size, and the use of prospectively collected data on several risk factors for breast cancer. Potential limitations arise from the use of a self‐administered questionnaire, which is more prone to response bias, and the possibility of unmeasured and residual confounding, which cannot be completely ruled out. In particular, we did not have information on physical activity, which is known to decrease breast cancer risk. Moreover, participants in a screening program may be not completely representative of the general population, having healthier lifestyle habits and being more health conscious than women who did not undergo the screening. However, the associations observed in the present study are generally consistent with the literature, thus reassuring against any major differential misclassification.

Thus, our study confirms the role of major recognized risk factors for breast cancer—including age at menarche, age at first pregnancy, alcohol drinking, and BMI (with a differential role according to menopausal status)—which are used in predictive models to estimate the individual absolute risk to develop breast cancer 20, 21. In addition, we observed an increased risk associated with a higher mammographic density. This is the first study that assessed these associations in an Italian population undergoing a mammographic screening. These findings will be helpful to develop a predictive model for breast cancer risk, focused on the Italian population, providing the basis for a stratification of the population according to different levels of risk, in order to offer differentiate screening procedure and timing.

## Availability of Data and Materials

The datasets are not available as they belong to the Agency for Health Protection of the Province of Milan.

## Conflict of Interest

None declared.

## References

[1] Torre, L. A. , F. Bray , R. L. Siegel , J. Ferlay , J. Lortet‐Tieulent , and A. Jemal . 2015 Global cancer statistics, 2012. CA Cancer J. Clin. 65:87–108. 25651787 10.3322/caac.21262

[2] Kolonel, L. N. 1980 Cancer patterns of four ethnic groups in Hawaii. J. Natl Cancer Inst. 65:1127–1139. 6933244

[3] Ziegler, R. G. , R. N. Hoover , M. C. Pike , A. Hildesheim , A. M. Nomura , D. W. West , et al. 1993 Migration patterns and breast cancer risk in Asian‐American women. J. Natl Cancer Inst. 85:1819–1827. 8230262 10.1093/jnci/85.22.1819

[4] Collaborative Group on Hormonal Factors in Breast . 2012 C. Menarche, menopause, and breast cancer risk: individual participant meta‐analysis, including 118 964 women with breast cancer from 117 epidemiological studies. Lancet Oncol. 13:1141–1151. 23084519 10.1016/S1470-2045(12)70425-4 PMC3488186

[5] Colditz, G. A. , H. J. Baer , and R. M. Tamini . 2006 Breast Cancer Pp. 995–1012 in Schottenfeld D. and Fraumeni J. F. Jr, eds. Cancer epidemiology and prevention, 3rd ed. Oxford Univ. Press, New York, NY.

[6] Hankinson, S. , R. Tamimi , and D. Hunter . 2008 Breast cancer Pp. 403–445 in Adami H.‐O., Hunter D. and Trichpoulos D., eds. Cancer epidemiology, 2nd ed. Oxford Univ. Press, New York, NY.

[7] Collaborative Group on Hormonal Factors in Breast Cancer . 2001 Familial breast cancer: collaborative reanalysis of individual data from 52 epidemiological studies including 58,209 women with breast cancer and 101,986 women without the disease. Lancet 358:1389–1399. 11705483 10.1016/S0140-6736(01)06524-2

[8] Bagnardi, V. , M. Rota , E. Botteri , I. Tramacere , F. Islami , V. Fedirko , et al. 2015 Alcohol consumption and site‐specific cancer risk: a comprehensive dose‐response meta‐analysis. Br. J. Cancer 112:580–593. 25422909 10.1038/bjc.2014.579 PMC4453639

[9] World Cancer Research Fund/American Institute for Cancer Research 2007 Food, nutrition, physical activity, and the prevention of cancer: a global prospective. World Cancer Research Fund/American Institute for Cancer Research, Washington, DC.

[10] Ferrari, P. , S. Rinaldi , M. Jenab , A. Lukanova , A. Olsen , A. Tjonneland , et al. 2013 Dietary fiber intake and risk of hormonal receptor‐defined breast cancer in the European Prospective Investigation into Cancer and Nutrition study. Am. J. Clin. Nutr. 97:344–353. 23269820 10.3945/ajcn.112.034025

[11] Aune, D. , D. S. Chan , A. R. Vieira , D. A. Rosenblatt , R. Vieira , D. C. Greenwood , et al. 2012 Fruits, vegetables and breast cancer risk: a systematic review and meta‐analysis of prospective studies. Breast Cancer Res. Treat. 134:479–493. 22706630 10.1007/s10549-012-2118-1

[12] Cao, Y. , L. Hou , and W. Wang . 2016 Dietary total fat and fatty acids intake, serum fatty acids and risk of breast cancer: a meta‐analysis of prospective cohort studies. Int. J. Cancer 138:1894–1904. 26595162 10.1002/ijc.29938

[13] Bernstein, L. 2002 Epidemiology of endocrine‐related risk factors for breast cancer. J. Mammary Gland Biol. Neoplasia 7:3–15. 12160084 10.1023/a:1015714305420

[14] MacMahon, B. , D. Trichopoulos , J. Brown , A. P. Andersen , P. Cole , F. deWaard , et al. 1982 Age at menarche, urine estrogens and breast cancer risk. Int. J. Cancer 30:427–431. 7141738 10.1002/ijc.2910300408

[15] McPherson, K. , C. M. Steel , and J. M. Dixon . 2000 ABC of breast diseases. Breast cancer‐epidemiology, risk factors, and genetics. BMJ 321:624–628. 10977847 10.1136/bmj.321.7261.624 PMC1118507

[16] Masala, G. , D. Ambrogetti , M. Assedi , B. Bendinelli , S. Caini , and D. Palli . 2017 Mammographic breast density and breast cancer risk in a Mediterranean population: a nested case‐control study in the EPIC Florence cohort. Breast Cancer Res. Treat. 164:467–473. 28478611 10.1007/s10549-017-4274-9

[17] Willett, W. C. 2001 Diet and breast cancer. J. Intern. Med. 249:395–411. 11350564 10.1046/j.1365-2796.2001.00822.x

[18] Zhang, S. , D. J. Hunter , S. E. Hankinson , E. L. Giovannucci , B. A. Rosner , G. A. Colditz , et al. 1999 A prospective study of folate intake and the risk of breast cancer. JAMA 281:1632–1637. 10235158 10.1001/jama.281.17.1632

[19] Michels, K. B. , A. P. Mohllajee , E. Roset‐Bahmanyar , G. P. Beehler , and K. B. Moysich . 2007 Diet and breast cancer: a review of the prospective observational studies. Cancer 109:2712–2749. 17503428 10.1002/cncr.22654

[20] Gail, M. H. , L. A. Brinton , D. P. Byar , D. K. Corle , S. B. Green , C. Schairer , et al. 1989 Projecting individualized probabilities of developing breast cancer for white females who are being examined annually. J. Natl Cancer Inst. 81:1879–1886. 2593165 10.1093/jnci/81.24.1879

[21] Pfeiffer, R. M. , Y. Park , A. R. Kreimer , J. V. Jr Lacey , D. Pee , R. T. Greenlee , et al. 2013 Risk prediction for breast, endometrial, and ovarian cancer in white women aged 50 y or older: derivation and validation from population‐based cohort studies. PLoS Med. 10:e1001492. 23935463 10.1371/journal.pmed.1001492 PMC3728034

